# Barriers and enablers to implementing multiple stroke guideline recommendations: a qualitative study

**DOI:** 10.1186/1472-6963-13-323

**Published:** 2013-08-19

**Authors:** Annie McCluskey, Angela Vratsistas-Curto, Karl Schurr

**Affiliations:** 1 Discipline of Occupational Therapy, Faculty of Health Sciences, The University of Sydney, New South Wales, Australia; 2 Physiotherapy Department, Bankstown Lidcombe Hospital, New South Wales, Australia

**Keywords:** Translational research, Implementation, Quality improvement

## Abstract

**Background:**

Translating evidence into practice is an important final step in the process of evidence-based practice. Medical record audits can be used to examine how well practice compares with published evidence, and identify evidence-practice gaps. After providing audit feedback to professionals, local barriers to practice change can be identified and targetted with focussed behaviour change interventions. This study aimed to identify barriers and enablers to implementing multiple stroke guideline recommendations at one Australian stroke unit.

**Methods:**

A qualitative methodology was used. A sample of 28 allied health, nursing and medical professionals participated in a group or individual interview. These interviews occurred after staff had received audit feedback and identified areas for practice change. Questions focused on barriers and enablers to implementing guideline recommendations about management of: upper limb sensory impairments, mobility including sitting balance; vision; anxiety and depression; neglect; swallowing; communication; education for stroke survivors and carers; advice about return to work and driving. Qualitative data were analysed for themes using theoretical domains described by Michie and colleagues (2005).

**Results:**

Six group and two individual interviews were conducted, involving six disciplines. Barriers were different across disciplines. The six key barriers identified were: (1) Beliefs about capabilities of individual professionals and their discipline, and about patient capabilities (2) Beliefs about the consequences, positive and negative, of implementing the recommendations (3) Memory of, and attention to, best practices (4) Knowledge and skills required to implement best practice; (5) Intention and motivation to implement best practice, and (6) Resources. Some barriers were also enablers to change. For example, occupational therapists required new knowledge and skills (a barrier), to better manage sensation and neglect impairments while physiotherapists generally knew how to implement best-practice mobility rehabilitation (an enabler).

**Conclusions:**

Findings add to current knowledge about barriers to change and implementation of multiple guideline recommendations. Major challenges included sexuality education and depression screening. Limited knowledge and skills was a common barrier. Knowledge about specific interventions was needed before implementation could commence, and to maintain treatment fidelity. The provision of detailed online intervention protocols and manuals may help clinicians to overcome the knowledge barrier.

## Background

### Translating evidence into practice

Translating evidence into practice, also known as implementation, is an active process involving individuals, teams and organisations [1]. Knowledge translation is an essential phase of evidence-based practice which is challenging, as this phase often involves changes in knowledge, attitude and behaviour. It cannot be assumed that an intervention which has demonstrated a positive effect, been described in a journal and recommended in a clinical guideline will be translated into practice [2]. Nor can it be assumed that the majority of people with a health condition will routinely receive that intervention [1].

Barrier identification is an important step in the process of knowledge translation [3]. As with quality improvement, clinical audits of practice may be conducted and audit feedback presented to staff [1], with opportunities provided for discussion of practice gaps. Evidence-practice gaps may arise because of systemic, team or individual barriers to change. Barriers may include lack of knowledge and skills, negative or out-dated attitudes, or inefficient systems [4]. Some of these barriers may also be enablers. For example, a senior clinician may reject a recommended practice or conversely act as an opinion leader [5]. While it is possible to anticipate some barriers, assumptions should not be made about which barriers affect a team or health service [6]. In addition, assumptions should not be made that barriers will be the same across disciplines.

### Translating stroke guideline recommendations into practice

Australian clinical guidelines for stroke management [7] aim to assist clinical decision-making and promote evidence-based care. Yet guidelines contain multiple recommendations which compete for a clinicians’ attention. For example, recommendation 6.3.4 (page 18) states that patients with difficulty walking should be given the opportunity to undertake as much repetitive practice as possible, which may involve using a mechanically-assisted device such as a treadmill. To implement this recommendation, physiotherapists may need to purchase a treadmill, learn how to operate the device confidently and persuade patients to trial the equipment. Assuming that clinicians agree with, and accept these recommendations, major changes in practice are often required.

Implementation of guideline recommendations may start by conducting a baseline medical record audit, to monitor practice. Clinical audits are commonly used for quality improvement, and the process is familiar to many clinicians. Feedback can then be provided to clinicians about audit findings. Audit feedback has been shown to influence practice [8]. A national audit of stroke services is conducted every two years in Australia. Subsequent audit reports provide valuable feedback about practice and allow benchmarking and comparison of like-services [9-12].

Audits of medical records are often conducted by individual stroke services between national sentinel audits. Staff on our stroke unit conducted three medical record audits between 2009 and 2011 to determine how much screening, assessment and intervention were being provided. An audit checklist was developed which included recommendations from the most recent stroke guidelines. A retrospective consecutive sample of 15 files of patients admitted to the stroke unit was audited in November 2009. The audits were conducted by the authors and additional staff on the unit. The audit revealed a number of practice areas where compliance with guideline recommendations was low (less than 60% compliance). Practice areas which became the focus for change included: upper limb sensory impairments, mobility including sitting balance and treadmill training; vision; anxiety and depression; neglect; swallowing; communication; education of stroke survivors and carers; return to work; and return to driving. After completing the first audit and providing feedback to stroke unit staff, the next step was to discuss potential barriers and enablers to implementation [6,13]. The process of using audit and feedback to drive behaviour change, and the outcomes will be described in a companion publication.

### Common barriers to implementation of stroke guideline recommendations

Barriers have been identified and reported for several areas of stroke care. These barriers include lack of resources, knowledge and skills, lack of motivation to change, professional’s beliefs about their capabilities, unhelpful attitudes about a guideline recommendation and role identity issues [14-20].

Professional’s beliefs about their capabilities have been reported as one key barrier to implementing recommendations [14,16,20]. For example, Canadian physical therapists lacked confidence in their ability to appraise and apply stroke research [16] recommended in guidelines. Occupational therapists in two Australian stroke services lacked confidence to take patients into the community for escorted outings and travel training [14], as recommended in national guidelines.

Attitudes to, and beliefs about, providing an intervention were a barrier in other studies, as well as beliefs about the original research. For example, Australian medical, nursing and allied health professionals reported concerns about patient safety and the impact on their workload when asked to implement protocols for fever, hyperglycaemia and swallowing management [15]. In that same study, there was a reluctance to accept evidence-based protocols for early management of swallowing problems using nasogastric feeding; that reluctance reduced compliance with the protocols [19].

Limited knowledge and skills represent a third barrier to implementing guideline recommendations in stroke rehabilitation [14-18,20]. In one Australian study, occupational therapists and physiotherapists reported a lack of knowledge about the evidence for providing escorted outings to people with stroke, to promote community participation [14]. Some stroke professionals in Canada reported difficulty appraising research and implementing some guideline recommendations [20], while others felt they possessed the necessary skills (and tools) to screen for depression [21]. Thus there can be differences across sites and disciplines. British occupational therapists wanted training to improve their confidence when conducting depression screening, particularly when screening patients with suicidal ideation [22].

Reduced motivation to change and implement a recommended practice is another known barrier [15,18]. For example Canadian occupational therapists reported low motivation to implement recommended neglect management [18]. In Australia, health professionals were resistant to implementing guideline recommendations for managing fever, hyperglycaemia and swallowing in acute stroke [15].

Limited resources is one of the most commonly reported barriers to implementing stroke guideline recommendations including lack of equipment, time and staff [14,15,17-20]. For example, equipment and time are necessary for implementation of neglect training [18]. The importance of allocating dedicated work time cannot be overstated; clinicians need to read and interpret original research [20] to understand what they must ‘do’ when implementing a recommendation.

Finally, difficulty accepting that a treatment is part of a discipline’s role is a sixth barrier to implementation of guideline recommendations. For example some professionals may not identify that a particular intervention is part of their role. Stroke professionals in Australia were concerned about blurring of professional boundaries related to management of fever, hyperglycaemia and swallowing after stroke [15]. Some occupational therapists and physiotherapists in Australia did not identify outdoor journey training as part of their role, reducing their compliance with guideline recommendations and the evidence [14]. On the contrary, Canadian allied health professionals generally perceived depression screening to be part of their role when a small sample of 19 staff were surveyed [21]. Similarly, in England, occupational therapists were keen to assume a role screening patients for depression in the absence of an on-site clinical psychologist, since therapists already screened stroke patients for cognitive impairments [22].

In summary, the process of identifying then targetting barriers is known to be important for successful knowledge translation. Failure to anticipate problems and barriers may results in little or no practice change. Barriers (and some enablers) have been reported to implementing stroke guideline recommendations in acute care and some areas of inpatient rehabilitation. While it is important to build on this existing knowledge, attitudes, skills and resources are likely to be different across settings, disciplines and countries. Limited research has been published about barriers facing Australian inpatient rehabilitation staff. Furthermore, much of the published data were generated from surveys, rather than in-depth interviews which can provide rich data and examples.

To help local professionals implement multiple stroke guideline recommendations, we engaged in a process to identify local barriers and enablers, informed by this prior research. We needed to determine what health professionals knew about the published research in the guidelines (knowledge), if they felt the research was strong enough to justify practice change (attitudes and intentions) and how able they felt to implement the specific recommendations and interventions with patients (skills and capabilities). The methods which we describe for obtaining the in-depth data, and the findings, should be informative for other stroke services.

### Aims of the study

The aim of this study was to identify local barriers and enablers to implementation of multiple guideline recommendations at one Sydney metropolitan stroke unit. These barriers were then targeted through regular coaching, audit and feedback to facilitate practice change as part of a broader long-term project.

## Methods

### Design

A qualitative study design was used to explore experiences, attitudes, knowledge and behaviour, and possible reasons for any evidence-practice gaps. The primary method of data collection was semi-structured focus group interviewing, with the option of an individual interview [23]. The aim of the group interviews was to stimulate interaction, encourage participants to respond and react to each other, and compare experiences [24]. Participants were allied health, nursing and medical professionals, employed at one stroke unit in Sydney, Australia. Ethical approval to conduct the study was obtained from a local area health service (Ref No. 2009/012).

### The sample

Three allied health disciplines were initially invited to participate (occupational therapy, physiotherapy and speech pathology). Ethics approval was extended to include additional disciplines involved in stroke patient care (nursing, orthoptics and medicine). Medical sub-specialties included geriatricians, rehabilitation specialists and neurologists, as well as registrars in training. These team members all worked closely together on the stroke unit, meeting weekly for case conferences, and each weekday morning for nursing handover. A total of 28 health professionals from the one site were recruited and interviewed. Written informed consent was obtained from each participant before they were attended their focus group.

### Interview procedures

Most participants (n = 26) were interviewed in discipline-specific groups, containing up to six people. Two orthoptists were interviewed individually, as neither worked onsite simultaneously. Interviews took up to one hour, and were conducted onsite at the hospital, between July and October 2010.

Interviews were moderated by the first and/or second author (AM/AV). The second person managed the audio-recordings, and kept detailed notes about the order of speakers and quotes [25]. The interviews with medical staff were not audio-recorded due to equipment difficulties during the group, and/or participant preference. Instead, in-depth handwritten notes were taken concurrently by the second researcher, and used for analysis.

#### The researchers

AM is an occupational therapist and health researcher with 30 years of clinical experience mostly in stroke rehabilitation. AM was not employed by the area health service, and did not work on a day-to-day basis with any of the participants. AV has an occupational therapy and research background, with 11 years of clinical experience in acute care and rehabilitation. AV was employed by the local health district and had contact with some of the participants as a research project manager. Most of the participants knew the interviewers personally or by reputation, because of their clinical and/or research backgrounds. As the researchers’ roles and positions could influence what participants said, they were advised that judgments would not be made about what the participants said or knew.

#### Focus of the groups and interviews

Questions focused on delivery of evidence-based treatments previously nominated by each discipline for practice improvement. Nominated treatment areas included management of upper limb sensation, neglect, sitting balance, treadmill training, swallowing, communication and education of patients and carers (see Table 1). The focus of each group interview was therefore slightly different. Questions posed during the medical staff group interviews focused primarily on the management of anxiety and depression, return to work, sexual functioning and driving. With orthoptists, questions focussed primarily on the management of vision impairments and neglect.

**Table 1:** Nominated areas for practice improvement based on national stroke guideline recommendations

***Discipline***	***Nominated area***
**Physiotherapy**	Improve the routine delivery and documentation of:
	• Sitting balance training to eligible patients
	• Treadmill training with harness support to eligible patients
**Occupational therapy**	Improve screening, assessment and intervention of/for:
	• Upper limb *sensory* deficits to eligible patients
	• Neglect to eligible patients
**Speech pathology**	Improve documentation of assessment, and intervention provided to eligible patients with:
	• Communication disorders including aphasia
	• Swallowing impairments
	Improve delivery and documentation of education provided to eligible patients and carers about:
	• Aphasia
	• Alternate methods of communication
	Periodic review of the severity of communication impairment.
**Nursing**	Improve delivery of education to eligible patients and family/carers
**Orthoptics**	Improve documentation and assessment of vision
**Medicine**	Improve documentation and management of:
	• Anxiety and depression for eligible patients
	• Return to work advice for eligible patients
	• Return to driving advice for eligible patients

Interview questions were developed by the first author. Questions were designed to elicit responses about factors that might help or hinder the uptake of each intervention.

#### Interview schedule

After first describing their discipline, years since graduation, and experience working with people following stroke, knowledge of the evidence was explored. The interviewer described the guideline recommendations, then enquired about group knowledge of these recommendations. Next, evidence-practice gaps or areas with lower compliance were discussed, based on guideline recommendations and audit findings. Reasons for these gaps were explored. Participants were encouraged to reflect, share, compare and react to group interactions [24]. Barriers and enablers to change were discussed. Possible solutions or ways forward were identified. Prompt questions were used to enquire about knowledge and skills, staffing, physical resources, assessment, screening and report writing systems and treatment routines. Group members explored their beliefs, attitudes and routines. The theoretical domains described by Michie and colleagues [4] were used to guide questions and data collection as reported in an earlier study by the first author [14].

### Data analysis

Four of the six group interviews, and two individual interviews were transcribed. In-depth handwritten notes from the two medical group interviews were typed up for use during analysis. Data analysis began after the first group meeting, and continued over seven months [26]. Participant statements were coded using the 12 conceptual domains described by Michie and colleagues as the guiding conceptual framework or theory [4]. This theory is intended for use by researchers who are exploring behaviour change, particularly barriers to evidence implementation. The theory, now referred to as the Theoretical Domains Framework, has recently been refined and includes 14 domains [27]. However the revised framework was not available at the commencement of this study.

Statements obtained during interviews were initially allocated to one or more category of the framework. For example, the statement *“There was a patient of mine (that) I would have never put them anywhere near it (the treadmill). I would have argued that it wasn’t good for them”* was placed in two categories: ‘beliefs about capabilities’ and ‘beliefs about consequences’. Statements were then allocated to the one category that best reflected the content topic. All quotes could be mapped to the framework.

Tables were generated that contained distilled summaries of participant experiences about barriers and enablers. This process was influenced by our original aim (to identify barriers and enablers which could be strategically targeted, with which behaviour change interventions).

## Results

Six group, and two individual interviews, were conducted with a total of 28 participants (see Table 2). Participants represented six disciplines, the majority of whom were medical professionals, occupational therapists or physiotherapists (n = 22; 79%). Participants were mostly female (88%). Demographic data are presented in Table 3.

**Table 2:** Focus group and individual interviews conducted by discipline

***Discipline***	***n (%)***	***Focus group interviews***	***Individual interviews***
Medicine	12 (43)	2	0
Occupational Therapy	5 (18)	1	0
Physiotherapy	5 (18)	1	0
Speech Pathology	2 (7)	1	0
Nursing	2 (7)	1	0
Orthoptics	2 (7)	0	2
**TOTAL**	**28**	**6**	**2**

**Table 3:** Demographic characteristics of health professionals (n = 28)

***Demographic variable***	***n***	***%***
**Discipline (n = 28)**		
Doctor	12	(43)
Occupational Therapist	5	(18)
Physiotherapist	4	(14)
Speech Pathologist	2	(7)
Registered Nurse	2	(7)
Orthoptist	2	(7)
Therapy Assistant	1	(4)
Gender (n = 16) *		
Female	14	(88)
Male	2	(12)
Clinical experience *(yrs) *(n = 16)*		
0–5	9	(56)
6-10	1	(6)
11–15	2	(13)
>15	4	(25)
Experience working in stroke *(yrs) *(n = 16) *		
0-5	9	(56)
6-10	4	(25)
11-15	0	(0)
>15	3	(19)

Note. * Data missing for medical professionals.

### Barriers and enablers

Factors that participants identified as barriers and enablers to practice change are presented in the following pages. There were six primary domains or categories of barrier; some were also enablers. For example, while one individual might believe they were unable to deliver a therapy due to lack of time or skills, another person might feel confident and able.

The first three categories of barrier were: (i) Beliefs about capabilities; (ii) Beliefs about consequences; and (ii) Memory and attention. These domains were discussed often by participants as potential or actual barriers to implementing stroke rehabilitation. These domains will be discussed first followed by three less dominant but important domains: (i) Knowledge and skills; (ii) Motivation, intention and goals; and (iii) Resources.

In addition to knowledge and skills, motivation, intention and goals, the domain ‘Resources’ was identified often by participants as an enabler to implementing evidence-based practice.

### Beliefs about capabilities

This domain refers to attitudes or beliefs about clinician’s individual ability, and the ability of their discipline to provide an intervention, assessment or test. This domain also includes beliefs about their ability to encourage patients to participate, and the ability of patients to participate in an intervention.

Physiotherapists described difficulties using treadmill training to improve the ambulation of stroke patients. First, they discussed the physical demands of this intervention. Second, they were concerned about having to stay with patients throughout their treadmill session. Due to safety concerns, they could not concurrently spend time with other patients in the gym while an individual patient was on the treadmill.

“We …[usually] move between patients…being stuck continuously with somebody for half an hour of treadmill …it’s a bit difficult…you can’t go and change what other people in the gym are doing” (PT1)

“…it’s physically quite demanding (treadmill training) …half an hour of assisting someone’s leg …I personally find [that] harder than overground walking” (PT4)

Some physiotherapists preferred to involve patients in overground walking rather than treadmill training because of the high physical demands on therapists.

“Other sites prefer treadmill training…they say it’s easier… less manual handling…we prefer overground [walking training]” (PT4)

“You couldn’t sustain it [treadmill training]… [because] you’d get a sore back” (PT2)

The physical ability of patients was another reported barrier. One physiotherapist reported that some patients were not capable of participating in treadmill training. They did not offer the intervention to such individuals. Yet sometimes patients surprised them and could use the treadmill.

*“There was a patient [that] I would never have put anywhere near it [the treadmill]. I would have argued that it wasn’t good for them. And it actually finished up good*” *(PT4)*

“It made me go ‘oh my goodness! This person can continuously practice for half an hour’. And I had to stop them… I would have never have thought they could…” (PT4)

Occupational therapists did not use sensation assessments with some patients because of their beliefs about patient ability. Poor communication and cognitive impairments made the assessment difficult. Furthermore, these assessments were not available in many languages.

“…sensory assessment can be quite abstract… if we have patients with quite severe communication problems, it can be very difficult to assess [them]” (OT2)

Nursing professionals also reported concerns about their ability to provide patient and carer education due to medical complications, cognitive and emotional impairments. These factors affected the patient and carers’ ability to receive and understand information.

"Q: ‘*What things might make it [patient education] happen or not make it happen?’ A: How (medically) stable they [the patients] are to receive the information… Are they able to cope or cognitively get [receive/understand] the information” (RN1)*

Nurses used different strategies and ways of explaining to help patients better understand information.

“Patients… learn differently. You have to apply different things… explain things a certain way” (RN2)

Speech pathologists, like occupational therapists and nurses, believed that patient factors including language and education capabilities sometimes limited their practice options. Many stroke patients came from a non-English background, and had a low level of education. These patients had difficulty participating in a standardised aphasia screening assessment. Many aphasia tests were not developed nor validated for use with patients from non-English speaking or diverse cultural backgrounds. Communication assessments became much more difficult.

“One of the problems … [with aphasia assessments] is that visual problems can alter the outcomes… as well as low education level …. in this demographic, some [patients] have a lower level compared with other area health services” (SP2)

“Some of those higher level language tasks in those [aphasia] tests tend to be quite culturally specific… [When] you’re asking somebody to finish a standard sentence in Australian English or American English… it doesn’t work for somebody of an Arabic background because they don’t know the context of the question…” (SP1)

In summary, individual clinician beliefs about their ability to conduct a test or deliver an intervention and beliefs about patient abilities were sometimes a barrier to delivering evidence-based practice.

### Beliefs about consequences

This category refers to clinician’s beliefs about the consequences of providing, or not providing an assessment or intervention. Beliefs that a treatment might produce adverse outcomes reduced the use of some therapies such as treadmill training. In other instances, the belief that therapists could make a difference and improve patient outcomes was enabling.

One physiotherapist believed that some patients did not exercise their affected leg adequately on the treadmill. This therapist preferred to use overground walking with patients in the gym, to avoid this potentially negative consequence.

“They can often get away without using their affected leg all that much [on the treadmill]” (PT1)

The same physiotherapist weighed up the consequences of delivering an evidence-based therapy such as treadmill training with very weak patients, when other types of training might produce better outcomes.

*“ If they’re that dependent, then we actually think it’s more worthwhile for them to be pushing on a tilt table, or doing sit-to-stand against a wall where they’re being really forced to use their intact leg rather than being put in a harness”* (PT1)

Speech pathologists were concerned about the consequences of wrongly interpreting results from standardised aphasia assessments, when used with non-English speaking or visually impaired people. These professionals wanted to use the ‘best’ test possible. However, these assessments had not been validated for use with non-English people. Therapists were concerned that language and vision problems would alter test scores.

“We’ve got a high non-English speaking population here, which means a lot of validated [aphasia] tests may not be that valid anyway [when] used [with] an interpreter” (SP1)

A nursing professional reported that some patients did not understand verbal information about their disease, medications or rehabilitation when delivered by some treating doctors. Such misunderstandings could have long term negative consequences for the patient’s health.

A medical doctor believed that screening patients for anxiety and depression was unnecessary. Yet this process was recommended as best practice in the Australian national stroke guidelines. That doctor did not foresee any negative consequences of ignoring the guidelines. They also believed that a positive result on an anxiety or depression screening tool did not always warrant treatment. More often it seemed that the doctors based their provisional diagnosis on clinical judgement and advice from the rehabilitation team. If a patient was suspected of having anxiety or depression only then would they be referred for a psychological review.

Another doctor worried about embarrassing patients if he asked about sexual activities, particularly patients from culturally diverse backgrounds. He avoided discussing the topic contrary to guideline recommendations. Another doctor avoided the topic for other reasons. He believed that sexual activities were less important to stroke patients because of their age.

“I tend to talk about sex with MS patients [people with multiple sclerosis] because they’re younger…and have spinal cord involvement…..often they will initiate discussion about sex….but stroke patients ….they tend to be older and ….might be embarrassed if we asked about that [sexual functioning]…particularly patients from other cultural groups here at this hospital” (MD2)

“I don’t routinely discuss sex with my patients unless they raise it….in the past, very few [patients] have asked about it” (MD1)

In summary, beliefs about the negative (or positive) consequences of using evidence-based practice affected the behaviour of most professionals. Areas of practice which were influenced include walking retraining, aphasia screening, delivery of stroke education and information including advice about resuming sexual activities and screening for anxiety and depression. These beliefs could be a barrier to evidence-based stroke rehabilitation.

### Memory and attention

This category refers to systems and prompts that reminded clinicians to deliver an intervention, or conversely, prompts that were absent and resulted in failure to act. Factors that made clinicians decide to act or not included competing tasks and priorities, time constraints and documentation systems.

Physiotherapists did not have an effective system in place that prompted the routine delivery of sitting balance and treadmill training to suitable patients. One physiotherapist said: *“It’s not part of my usual thinking”.*

Another physiotherapist talked about forgetting to provide sitting balance training to patients who could stand and walk. They knew that evidence existed supporting the use of sitting balance training to improve the performance of standing up, but forgot this fact when busy.

“… we go straight into sit to stand… and standing and walking, and we don’t then go back to doing it [sitting balance]” (PT1)

Several physiotherapists knew they were forgetting to record patient practice in the medical records.

“I think that’s [sitting balance] definitely one that we’re not documenting enough. That came out recently [from a file audit], that a number of people that should be getting it, were not” (PT3)

“A lot of practice sheets hadn’t been put into the [patient] notes…There was a pile on desks that I collected and put into medical records” (PT2)

Speech pathologists routinely assessed communication, but often forgot to provide or document interventions which were recommended in the stroke guidelines.

“We don’t necessarily [document] review of severity [of communication impairment]… it often gets missed” (SP2)

Nursing professionals provided education to patients and carers during a weekly education group. However, they often forgot to report this intervention in the medical records. One nurse knew that they had educational DVDs about stroke, but would often forget to offer them to patients and carers.

“They [educational DVDs] were available at some stage but I think if they’re locked up, we’re going to forget to offer them” (RN)

Orthoptists discussed the management of visual impairments and provision of education to patients. They routinely provided education but again, this intervention was not always documented in the medical records.

“I’ll admit this…I do not write down in the file when I have given education to the patient” (Orth2)

“It wasn’t that it wasn’t being done [education] it was that we weren’t documenting that information in the file” (Orth1)

In summary, most disciplines reported forgetting to provide and/or record some interventions or assessments. They felt that better recording systems would prompt them to practice differently. Thus, an improved recording system was one possible solution to target the memory and attention barrier.

### Knowledge and skills

Limited knowledge and skills was a barrier for some disciplines, but an enabler to others, to providing evidence-based assessment and intervention. Disciplines such as physiotherapy and speech pathology seemed to know the research well, and how to deliver named interventions. That knowledge became an enabler to change.

Physiotherapy staff knew the research about sitting balance and treadmill training. They were aware of guideline recommendations that supported the use of treadmill training for patients with severe mobility impairment. Clinical protocols were already available within the department and many physiotherapists had the skills to provide the interventions to appropriate stroke patients.

“[Researcher X] did a study where people were allocated to either treadmill or overground walking…for half an hour a day… the people in the treadmill group, 17% or 18% more achieved independent walking” (PT1)

“Q: With seated reaching…do you know how to do that already?” A:“We’re very familiar with [researcher X’s] sitting balance research” (PT):

On the contrary, when occupational therapists talked about management of sensation and neglect they reported a knowledge and skills gap. They did not know where to start, what assessments or interventions to obtain, nor how to use these with patients.

“We don’t know an awful lot about it [sensation and neglect rehabilitation]. This is a good opportunity to learn…start to change our practice” (OT1)

“I was at XX [hospital]… for sensory retraining there, we used to do… stereognosis in a bag of rice…but that’s all we did… There was not much evidence to back up what we were doing” (OT4)

Speech pathologists knew about the guideline recommendations for managing swallowing and communication difficulties. They were also aware of other research about best-practice management of communication impairments. They demonstrated knowledge of various standardised tools used to screen for aphasia.

“The guidelines do say [that] 100%… everyone… should get a swallowing screen and everyone should get a communication screen” (SP2)

Nursing professionals felt they had a good knowledge of stroke but insufficient skills to teach patients and carers effectively.

“A majority of the nurses have been working in stroke longer than us on this ward together. So I think their knowledge and their skills are huge and they are stroke specialists” (RN1)

“Some of the staff have a very good knowledge base [about stroke in order to educate patients and carers] but they don’t put that into use” (RN2)

As previously reported, some medical professionals were uncomfortable discussing sexual activities with stroke survivors. One doctor did not know what to say, or how to advise patients who were keen to resume sexual activities.

‘I honestly don’t know what to say if a patient brings it up [the topic of sex]…. I’m not sure whether Viagra is Ok for patients to use or not’ (MD1)

Medical professionals acknowledged that depression and anxiety were important problems experienced by patients. They knew that there was robust evidence about the impact of these impairments on stroke outcomes but were not yet screening patients for depression and anxiety.

In summary, knowledge about research contained in the national stroke guidelines varied across disciplines. Not knowing the research, or how to implement a guideline recommendation was a barrier to the provision of evidence-based stroke care.

### Motivation, intentions and goals

This category refers to clinician’s motivation or intention to provide an evidence-based test or therapy. This category also refers to how much they wanted or needed to do a test or therapy and whether other priorities interfered with their intentions.

Occupational therapists struggled to complete all the necessary assessments and interventions in a working day. They intended to prioritise assessments and interventions which would produce the best patient outcome. Yet like many professionals they had difficulty fitting their assessments and intervention around other rehabilitation commitments.

‘There’s so many interventions that we need to do as OT’s and … we have to pick the one that’s going to have the biggest impact for the patient….balancing what’s going to be most effective and have the best outcomes for the patient?” (OT1)

“If we had a way of prioritising… ‘Yes, this (sensation) is the thing that’s impacting their fine motor ability’…. That would certainly be more motivational … we would have a focus on that for that patient” (OT1)

Speech pathologists used a prioritisation system that some felt was a barrier to routinely providing intervention for communication impairments.

“Our prioritisation is one of the reasons why we may not get to [do] an intervention as frequently as the guidelines say…the way we prioritise patients is very acute based. So sometimes when you’ve got more patients…communication patients go down the list” (SP2)

Some speech pathologists considered documentation about aspects of patient education to be a lower priority.

“Swallowing education…. that tends to be reasonably well documented. But … communication, it’s not something that we tend to see as an urgent thing to put in the notes …that you’ve done it (SP1)

Motivation to provide an intervention was an enabler for medical, nursing and allied health professionals. Some participants were keen to provide best practice.

“We decided as a group to focus on sensation and neglect …we thought this was a good opportunity to be able to learn and start to change our practice” (OT1)

“Maybe that’s something [treadmill training] we really should be doing …routinely with non-walkers” (PT1)

In summary, being motivated to provide evidence-based care was an enabler for some participants, however, sometimes other priorities got in the way.

### Resources

This domain refers to the presence or absence of resources such as staff, materials, space, time and the predictability of these more tangible resources.

When physiotherapists talked about providing treadmill training, they lamented the time and staff required to conduct each patient training session. A session took almost 45 minutes including preparation and usually required the presence of two physiotherapists.

…it’s [going to] take a second person to get them on the treadmill and once they’re on the treadmill, you’re stuck with them” (PT1)

“What about the treadmill?” (PT4) “What stops us using it?” (PT1): “I reckon it’s time more than anything” (PT2)

Occupational therapists and speech pathologists reported fluctuating staff levels, which affected the amount of intervention they could provide. Reduced staffing was an ongoing barrier to best practice throughout the study period. Occupational therapists had difficulty finding time to provide best-practice sensation rehabilitation in addition to their usual care. Speech pathologists were limited in how much communication training they could deliver for the same reason.

“The other thing about intervention with communication is… staffing levels dropping …with winter coming…” (SP2).

“Our capacity to do intervention for communication is a lot lower” (SP1)

Speech pathologists did not have enough written information to give to patients and carers about the management of swallowing and communication impairments. Of particular concern was the lack of information that could be easily understood by patients with aphasia. They also did not have standardised tools in the department to formally screen patients for aphasia.

“We don’t own them [validated aphasia tests]… They cost about $50-$100, so we just want to work out which one to buy” (SP2)

Nursing professionals did not have enough written educational materials to give to patients, including translated materials. They previously had videos to show to patients and carers, but these had gone missing. The cost of purchasing and replacing lost materials was a barrier to education. Weekly education sessions were delivered in the ward dining room, but transporting patients to this area could also be difficult. Limited availability of language interpreters was another reported problem when providing education to some patients and carers.

“The only way we’re offering it [education] at the moment is when interpreters are booked. So if OT gets an interpreter, that’s when they’re given the education. But… that’s probably once during the admission, if it happens at all” (RN1)

“The (educational) pamphlets in different languages…are not available at the moment….” (RN1)

“We can get some good ideas [re: educational material] and then look at cost as well… [cost] does come into it (RN1)

“Patients have got to be able to get to the dining room [for education sessions]… [mobility] can also be a barrier” (RN2)

Finally, orthoptists reported making time to assess patients, but had little time for treatment. Both they and the nursing professionals also reported difficulty accessing patients and carers for education sessions.

“You can see every patient, diagnostically speaking, but you haven’t got time to do treatment” (Orth2)

“You’re also fighting the other professions because the person’s in the gym, [or] they’re with the speechie… (Orth2)

Resources could also be an enabler. Physiotherapists had developed local protocols and had the necessary equipment for sitting balance and treadmill training. Occupational therapists had found some prism glasses which were one intervention they needed to provide as part of neglect retraining. Nursing professionals had purchased some educational DVDs and pamphlets on stroke. In summary, availability of staff, time and equipment varied across disciplines and impacted on clinician’s ability to provide evidence-based practice.

## Discussion

There were three key findings in this study. First, reported barriers and enablers were different across professions. Many barriers were expected, but some beliefs were not and may be more difficult to change. Second, gaps in knowledge and skill were common. Many therapists did not know what to do after reading a guideline recommendation. Third, participants identified strategies while reflecting during the interviews, which they could use to change practice. Finally, this study provides applied examples of the convergence between evidence, clinical judgement and patient values or circumstances [28].

### Different barriers across professions

The theoretical domains framework proposed by Michie and colleagues (2005) helped identify barriers and enablers which were present, and those which were absent. Using the framework was helpful during the interview process. Individual professionals and disciplines became clearer about which barriers needed to be addressed, who needed to work differently, and what type of behaviour change strategies might be helpful [27].

The interviews allowed time for therapists to systematically reflect on potential barriers affecting their practice or discipline, and behaviour change strategies that might be needed. For example, to improve knowledge about sensation and neglect rehabilitation, occupational therapists left the interviews recognising that they needed to obtain and read relevant journal articles, make contact with known experts in the field, purchase and trial equipment. Prompts were identified and welcomed which could improve attention to procedures such as depression screening or advice about return to driving. For example, one team member suggested introducing a ‘standing item’ of business to the weekly case conference, to prompt memory and action. They decided to check if driving has been discussed and documented for individuals who had driven pre-stroke.

Asking each discipline to select one or two focus areas for quality improvement worked well after barriers had been identified. We recommend this strategy when initially trying to improve practice and change behaviour. However, in the long term some guideline recommendations and some barriers are more important to address than others, with practice and policy implications. For example, underuse of swallowing screening, assessment and retraining may be considered to be of greater importance because of the risk of aspiration.

Some barriers and practice areas proved too challenging. One important practice area which none of the disciplines selected for improvement was sexuality. The Australian guidelines recommend that stroke survivors and their partners be offered the opportunity to discuss sexuality with an appropriate health professional and be offered written information addressing sexuality post stroke [7]. A recent national audit found that only 17% of Australian stroke patients received such advice (an improvement from 0% in 2009) [29]. Sexuality education and advice appear to be resistant to change. Many barriers exist for patients, carers and staff.

Several barriers to providing best practice sexuality advice were identified at our stroke unit, for staff and patients. There were unhelpful beliefs about the consequences of raising sexuality with patients, and gaps in knowledge and skill. If discussions are occurring, they were not being documented. The honest quotes from participants imply a need for skills training. Such training might include role playing with simulated patients and practice discussing sexuality, to improve communication, confidence and help change behaviour.

Role playing has been used as a behaviour change technique in primary care by Cane and colleagues [27]. These researchers helped general practitioners to rehearse the process of telling patients with acute low back pain that a plain film X-ray was unnecessary. Cane and colleagues also disseminated a DVD which presented ‘model’ responses if a patient repeatedly asked for a plain film X-ray to be completed. Implications for education from our research include the potential for a DVD to teach professionals how to better communicate about sexuality post-stroke. Sample scripts or narratives could be offered to replace the awkward silence that sometimes occurs. Such materials would be useful to many services.

### Addressing gaps in skill and knowledge

The skills and knowledge barrier to evidence-based rehabilitation is surprisingly common, with implications for graduate and entry-level education. Where intervention protocols existed, the therapists were often able to obtain and trial them. For example, the physiotherapists had participated in randomised trials of sitting balance and treadmill training and understood the protocols. They knew what to do. If they had not been involved in the original trials, they would have experienced similar barriers to other professionals. Occupational therapists in this study contacted a local expert who had presented a conference paper about neglect rehabilitation. The expert visited the unit and demonstrated how to use visual scanning. This consultation overcame the knowledge and skill barrier which arose because no written treatment protocol was freely available.

Other areas of practice which were difficult to implement because of a skills and knowledge barrier include sensation retraining, mental practice and constraint therapy to promote upper limb recovery. Many hours were spent working out ‘what to do’. Treatment protocols need to be more easily available when trials of effective intervention are completed. Protocols may include videos and photographs of procedures. One such example is the GRASP program (Graded Repetitive Arm Supplementary Program) for hand and arm rehabilitation, developed by Professor Janice Eng and colleagues in Canada [30]. Following publication of their trial, the research team prepared documents with photographs of the GRASP treatment protocol, with additional implementation grant funding. The procedures are freely available to stroke survivors and therapists at http://neurorehab.med.ubc.ca/grasp/. Implementation becomes easier when protocols are available to therapists. One research and policy implication is that triallists could be required to provide their treatment protocols freely to clinicians, when an intervention is found to be effective.

Screening patients routinely for the presence of depression was another practice affected by the skills and knowledge barrier, as well as beliefs and attitudes. Depression screening is an international challenge. Low compliance with guideline recommendations has been reported in England [22], Canada [21] and our stroke unit in Australia. Recent 2012 Australian audit data revealed that only 50% of stroke patients were screened or assessed for depression across over 100 hospitals [29]. Kneebone and colleagues (2010) in England have implemented behaviour change strategies to address this evidence-practice gap. They trained occupational therapists who volunteered to conduct routine depression screening, then tested their knowledge and skills. Next, they checked the medical records, to ensure fidelity and accuracy of screening procedures by participating therapists, and provided feedback. Similar training could be provided in Australia, with implications for education and practice.

### Convergence between evidence, clinical judgement and patient circumstances

Several examples were provided where therapists reported using clinical experience and knowledge of patient circumstances alongside published evidence. Speech pathologists knew that aphasia test results would be invalid if a patient could not speak or understand English. Consequently they chose not to conduct these tests on people who were unable to speak English. Physiotherapists weighed up the time taken to set patients on the treadmill with a harness and two therapists against the potential outcomes of using a much simpler therapy-sitting to standing training to improve leg strength. They kept patient outcomes and benefit in the front of their mind and thought carefully about time management in the busy ward setting. These examples have implications for professional education, and could be used to highlight patient circumstances that influence decision-making and compliance with guideline recommendations.

### Study limitations

As with all research, our study had limitations. First, only one site was involved. Findings are unique to that site and participating professionals. However, findings are likely to be useful to other professionals and stroke units with similar characteristics. Second, the study would have been strengthened by conducting a second round of interviews with staff.

A third limitation, but also a strength was the use of a theoretical framework to guide the interview schedule and data analysis. Use of this theory may have prevented categories from emerging which did not ‘fit’ those documented by Michie and colleagues (2005). However, the benefits of using this framework, including the efficiency with which interview data could be coded, in our view outweigh the limitations for busy clinical for education, practice, policy and future research.

### Implications for education, practice, policy and future research

Education implications are relevant to universities and professional associations, as well as peak bodies such as the Australian National Stroke Foundation. First, there was a need for communication training about sexuality post-stroke, possibly involving model scripts and narratives. Education needs also included ‘how to’ conduct routine depression screening, neglect and sensation training, and ‘how to’ deliver mental practice. Anecdotally, these skills and knowledge gap are known to be common across many services. Professional associations and the National Stroke Foundation are already collaborating to address these knowledge gaps.

Policy and practice implications include the need to target ‘high risk’ evidence-practice gaps, such as low compliance with swallowing screening, assessment and retraining. These practice gaps have implications for patient safety, due to the risk of choking and aspiration pneumonia.

There are at least two research implications from this study. First, more research is needed into behaviour change strategies that can, and do influence ‘difficult to shift’ practice areas such as sexuality education and depression screening after stroke. Second, triallists who develop effective rehabilitation interventions could be required to make their treatment protocols freely available to clinicians.

## Conclusions

Knowledge translation is an important final step in the process of evidence-based practice. This qualitative study describes the process of identifying barriers to implementing guideline recommendations in stroke rehabilitation. The interviews enabled professionals to identify areas in need of change, reflect on barriers, and how each practice area could be targeted with behaviour change interventions. Some practice areas generated interesting attitudinal barriers and beliefs. Our qualitative data add to the current body of knowledge about barriers in these more difficult practice areas, and may be informative for other teams.

## Competing interests

The authors declare that they have no competing interests.

## Authors’ contributions

The first author AM conceptualised and planned the study, writing of the manuscript, collection and analysis of data and writing of the manuscript drafts. The second author AV-C helped collect and analyse data and complete manuscript drafts. The third author KS advised on study design, and manuscript drafts. All authors read and approved the final manuscript.

## Pre-publication history

The pre-publication history for this paper can be accessed here:

http://www.biomedcentral.com/1472-6963/13/323/prepub
